# Work-Related Psychosocial Stress in Small and Medium-Sized Enterprises: An Integrative Review

**DOI:** 10.3390/ijerph17207446

**Published:** 2020-10-13

**Authors:** Elena Christina Schreibauer, Melina Hippler, Stephanie Burgess, Monika A. Rieger, Esther Rind

**Affiliations:** 1 Institute of Occupational and Social Medicine and Health Service Research, University Hospital Tuebingen, 72074 Tuebingen, Germany; elena.schreibauer@med.uni-tuebingen.de (E.C.S.); melina.hippler@med.uni-tuebingen.de (M.H.); stephanie.burgess@med.uni-tuebingen.de (S.B.); monika.rieger@med.uni-tuebingen.de (M.A.R.); 2 Interdisciplinary Division of Neuro-Oncology, University Hospital Tuebingen, 72076 Tuebingen, Germany

**Keywords:** small and medium-sized enterprises, work-related stress, psychosocial factors, work patterns, integrative review

## Abstract

*Background*: Work-related psychosocial stress can cause mental and physical illnesses resulting in high costs for the individual, the economy and society. Small and medium-sized enterprises (SMEs) employ the majority of the world’s workforce and often have fewer financial and human resources compared to larger businesses. The aim of this review is to summarize current knowledge on work-related stress in SMEs according to well-established guidelines categorizing psychosocial factors at work. *Methods*: A systematic database search was carried out in PubMed, PsycINFO, PSYNDEX and Business Source Premiere from March to June 2019, updated in January 2020. Data of included studies were analyzed and mapped into five themes: “work content and task”, “organization of work”, “social relations”, “working environment” and “new forms of work”. *Results*: After full-text screening, 45 out of 116 studies were included for data extraction. Studies were very heterogeneous and of varying quality, mostly applying a cross-sectional study design. Psychosocial factors in SMEs have been researched with a focus on the work patterns “work organization” and “work content and task”. *Conclusions*: This review underlines the need for more and better quality research of psychosocial factors in SMEs, particularly in relation to ongoing and new challenges in the workplace, including stressors related to the process of digitalization or the development of safe working conditions during the emerge of new infectious diseases.

## 1. Introduction

According to the International Labour Organization (ILO), 2.8 million workers die from work-related diseases each year and there are about 374 million non-fatal work-related injuries annually [1]. This results in human and economic costs of about 4% of the annual global gross domestic product [1]. Additionally, work-related psychosocial risks, comprising issues such as work-related stress, have been identified as significant risks in the field of occupational health and safety over the last two decades [2,3]. However, as of yet only a few countries have drafted specific regulations on psychosocial risks (e.g., the 2014 Belgian Royal Decree on the prevention of psychosocial risks at work or the Colombian Resolution 2646 on risk assessment and management of psychosocial hazards from 2008) or have implemented existing policies in their national occupational safety and health legislation [4,5]. Furthermore, there is little evidence whether and how these recommendations and policies can be implemented in a working environment with limited human and financial resources, including, for example, small and medium-sized enterprises (SMEs) [6]. A number of ILO conventions (e.g., Convention on Occupational Safety and Health, 1981 (No.155) and its accompanying recommendation (No. 164), the Occupational Health Services Convention, 1985 (No. 161) and its accompanying Recommendation (No. 171) and the Promotional Framework for Occupational Safety and Health Convention, 2006 (No. 187) and its accompanying Recommendation (No. 197)) provide an international framework for national occupational health and safety legislation [7]. The aim of this legislation is the prevention of work-related accidents and illnesses by minimizing the causes of hazards in the working environment in order to protect the physical and mental health of workers. Measures should also consider the relationship between humans and the working environment, including the matching of machinery, equipment, working time, work organization and work processes to the physical and mental capacities of workers. In Europe, work-related health issues and occupational diseases contribute considerably to absenteeism and incapacity to work with socioeconomic consequences for individuals, the economy and society [8,9]. In Germany, for example, sickness absence caused by mental disorders has increased significantly over the last 40 years from 2% to about 16% [10,11]. The estimates of total costs (direct and indirect) of mental health in the EU28 amount to 4.1% of the Gross Domestic Product (GDP) [9]. Therefore, work-related psychosocial demands, including job content, work intensity or the social environment, have gained political and scientific attention [12]. When psychosocial demands continuously exceed the resources and coping capacities of employees, this can result in perceived stress [13] and, as a long-term consequence, the development of mental disorders [14,15,16] as well as somatic or psychosomatic diseases [17,18,19]. In 2008, the European Union highlighted the need for action in the area of psychosocial risks (e.g., poor organization of work, lack of role clarity, lack of support from supervisors or colleagues) in order to reduce economic and social costs associated with work-related stress [12,20].

The Council Directive (89/391/EEC) on the introduction of measures to encourage improvements in the safety and health of workers at work [21] gives employers in the European countries the legal responsibility to prevent and reduce work-related risks. This comprises, for example, the adaption of workplaces with regard to physical risks (e.g., considering ergonomic aspects such as the choice of working equipment or production methods) as well as the prevention of psychological risks (e.g., work-related stress through a monotonous working environment). In Germany, this directive has been implemented in the Safety and Health at Work Act [22], which specifically obliges the employer to assess work-related health risks. This includes not only physical aspects of work (e.g., design and setup of the workstation or production methods), but explicitly mentions the requirement to assess “psychological stress at work”.

Regarding the implementation of the Safety and Health at Work Act in Germany, surveys in 2011 and 2015 [23] showed, that only about 53% of all companies had carried out risk assessments and less than 40% of them had assessed psychosocial risks. Compared to larger companies, the percentage of completed risk assessments in small and medium-sized enterprises (SMEs) in Germany is considerably lower: only 38% of SMEs indicated the completion of risk assessments and only 6% of SMEs the assessment of psychosocial risks [23]. This problem does not only occur in Germany but affects several states of the European Union, as European surveys (ESENER 2009, 2014 and 2019 [3]) with a special focus on emerging risks have shown [24]. This poor implementation among SMEs has been related to limited human and financial resources which may result in a relatively low priority towards occupational safety and health (OSH) management [6]. SMEs have, however, a large impact on the socioeconomic welfare of countries all over the world, as they represent the majority (>90%) of the non-financial business economy and employ over 60% of all workers [25,26,27,28]. As current research indicates that psychosocial demands in SMEs differ from those in large companies [29,30,31], several tools have been developed by European and German OSH institutions to support SMEs in implementing comprehensive risk assessments [32,33,34]. Nevertheless, the implementation of the legal requirements in SMEs has improved only slightly, and despite all efforts, the number of risk assessments of psychosocial factors carried out in German SMEs has hardly increased between 2011 and 2015.

In order to support enterprises with the development of instruments and interventions to reduce psychosocial risks, knowledge on relevant work-related psychosocial factors is essential. Between 2014–2017, the German Federal Institute for Occupational Safety and Health (BAuA) conducted several literature reviews to describe the scientific evidence regarding mental health in the working world [35]. Based on well-established theoretical models including the Job-Demand-Control-Model [36], the Effort-Reward-Imbalance-Model (ERI) [37] or the Organizational Justice (OJ) model [38], a framework was developed to categorize the results into four subject areas: “Working Task”, “Leadership and Organization”, “Working Time” and “Technical Factors”. The reviews did, however, not specifically focus on the situation of employers and employees working in SMEs. Furthermore, the Joint German Occupational Safety and Health Strategy (GDA) published recommendations for implementing psychosocial risk assessments listing five work patterns (i.e., work characteristics) that have been identified as primary stress factors in the workplace [39]:

- work content and task (e.g., job autonomy, completeness of tasks, emotional demands);
- organization of work (e.g., working time and processes);
- social relations (e.g., aspects of hierarchy, leadership and managerial abilities);
- working environment (e.g., physical factors, working equipment);
- new forms of work (e.g., spatial and temporal mobility).

To organize the results of this review, we used this classification because it comprises all of the psychosocial factors published by the BAuA [35] and integrates factors published by other well-established international frameworks (EU-OSHA (The European Agency for Safety and Health at Work) [12], WHO (World Health Organization) [40] and ILO [5]). Furthermore, the GDA-classification includes a systematic table and detailed examples of work-related psychosocial demands which provided a useful framework for a clear assignment of the studies identified in this review.

To the best of our knowledge, this review is the first to summarize and categorize the current evidence on work-related psychosocial demands with a specific focus on small and medium sized enterprises to identify gaps in current knowledge and provide a systematic overview of which psychosocial factors, outcomes and economic sectors have been considered to date. The subsequent research questions guided our review:

(1) What is the current state of knowledge on psychosocial demands in SMEs?
(2) Which outcomes and economic sectors have been examined?

## 2. Materials and Methods

### 2.1. Review Design

We conducted an integrative review according to the five-stage method described by Whittemore et al. [41]. To select relevant studies, we used the PEO criteria (population, exposure, outcome) which are applied frequently in evidence-based health research [42,43]. For our study, we defined the criteria as follows: P = workforce in SMEs (with SMEs defined according to the EU definition: number of employees < 250 [44]) and E = psychosocial demands as defined by the GDA [39]. We did not predefine any outcomes for the literature search because we did not want to limit our review to certain health or health-related outcomes.

### 2.2. Search Strategy and Study Selection

The literature search was carried out between March and June 2019 and updated in January 2020 with individually adapted search strings in medical (PubMed), psychological (PsycInfo, PSYNDEX) and economic (Business Source Premiere) databases. Additionally, we performed a hand search of the reference lists of the studies included. The search terms followed this general scheme: terms related to “workforce in SMEs” AND terms related to “psychosocial demands”. We also considered relevant Medical Subject Headings (MeSH)-Terms and search terms previously published [45]. Terms including “family business(es)” were excluded because this resulted in a considerable number of articles not relevant in our context. For example, many articles dealt with the financing of micro-enterprises or the family income situation in developing countries. The complete search strategy for PubMed and the applied search string is presented in Supplementary Table S1.

We included all types of peer-reviewed quantitative and qualitative studies as well as literature reviews relevant to the SME setting considering at least one of the psychosocial demands listed by the GDA [39]. Articles that did not report employment figures, but whose surveyed enterprises were designated as SMEs, were included if their SME definition complied with the European definition [44]. If multiple publications were based on the same dataset, all papers meeting the inclusion criteria were selected. We limited our search to articles published in German or English from January 2000 onwards, the beginning of the fourth industrial revolution promoting digital processes in the working environment [46,47] introducing new stressors to the workforce such as digitized performance monitoring or information overload [47,48]. Furthermore, we excluded study protocols as well as publications which tested and/or validated questionnaires on psychological demands or work-related stress. We also excluded reviews because we considered the original research and did not consider any kind of “grey literature”.

After the removal of duplicates, all studies were transferred to Rayyan [49], a free web application for systematic review screening. Two raters (E.C.S. and S.B.) screened titles and abstracts independently, according to the predefined inclusion and exclusion criteria. Articles that could not be judged by title and abstract were included in the full text screening, also independently executed by two raters (E.C.S. and M.H.). Lack of agreement was solved in consensus discussions with a third reviewer (E.R.).

### 2.3. Full-Text Analysis and Quality Assessment

A tabular scheme for data extraction (author, year, country, year of data collection, topic, study design, data collection methods, type of enterprise, sample size, industrial classification of the business, investigated psychosocial demands, outcomes, and data collection instruments) was compiled and used for data extraction. We used the Agency for Healthcare Research and Quality AHRQ study design algorithms [50] to classify the study design if it was not reported. The allocation of the studies to economic sectors was carried out according to the International Standard Industrial Classification of All Economic Activities (ISIC) [51]. We amended the classification of the BAuA-project [35] to categorize the outcome variables of the studies into these subcategories: general (work-related) stress outcomes, health, well-being, factors affecting cardiovascular health, mental health, musculoskeletal system, social relations, and business-related outcomes.

Different tools were used for critical appraisal: the Special Unit for Review Evidence (SURE) checklist for cross-sectional studies [52] the SURE-checklist of randomized controlled trials and other experimental studies [53], the SURE-Checklist for qualitative studies [54], and the Joanna Briggs Insitute (JBI)-Critical appraisal tool [55] for text and opinion articles to assess the quality of narrative reviews.

## 3. Results

### 3.1. Study Selection

**Figure 1:**
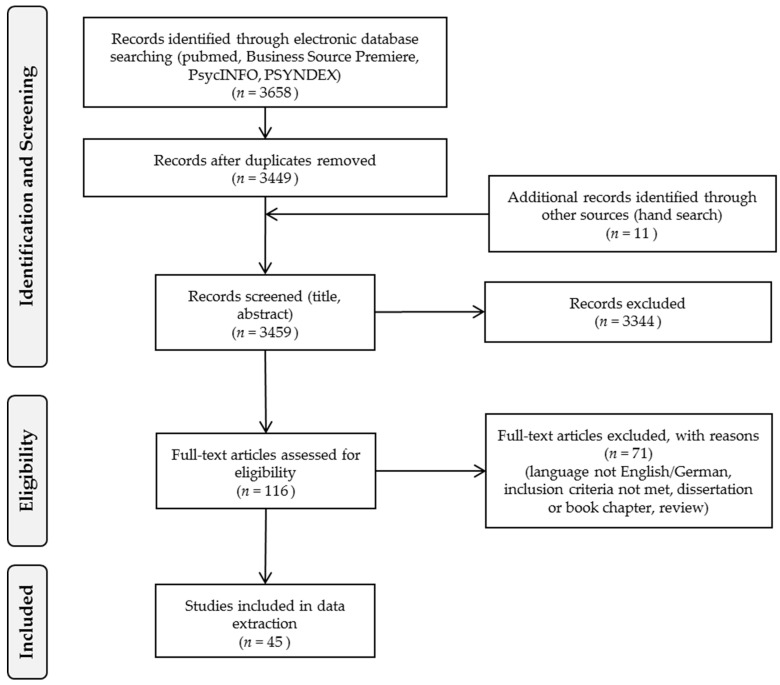
PRISMA Flow Diagram [56] on database search and study selection.

We identified 3658 studies through electronic data base searching (see PRISMA-flowchart [56] Figure 1). After the removal of duplicates, 3460 studies remained for title and abstract screening, including eleven articles detected by hand search. For the full-text-screening, 116 articles were eligible, of which 45 were included in the full text analysis (Figure 1). The first article suitable for this review was published in 2004. Since then, the annual number of publications was relatively low (<5/year) with a peak in 2018, when nine articles were published. Due to our main database search in the first half of 2019 (hand search update March 2020), it can be assumed that studies published in 2019 were not completely indexed at that time.

### 3.2. Sample Characteristics and Study Designs

The samples of the studies were very heterogeneous. The number of participants varied between seven and 23,000. Five studies included only SME-managers and enterprise owners [57,58,59,60,61]. Some studies used data from nationwide surveys that provided a large sample size and included a range of economic sectors and branches [29,30,62,63,64,65,66,67]. Other studies included only participants of a single enterprise [68,69,70,71]. A complete summary of the study characteristics can be found in Supplementary Table S2.

Most of the studies included were carried out in Europe (*n* = 24) and in Asian countries (*n* = 13). Other studies were conducted in Australia [57,72], the United States [73] and New Zealand [74]. Four studies from Japan [62,64,65,66] seemed to use data from the same survey but examined different outcomes. No studies from Africa or South America met our inclusion criteria, and four studies were transnational [61,75,76,77].

The majority of studies were cross-sectional (*n* = 37), analyzing a variety of psychosocial factors in the SME-setting, mainly using data from nationwide surveys or specifically designed questionnaires for particular companies or settings. We also identified qualitative studies (*n* = 2), narrative reviews (*n* = 3), and intervention studies (*n* = 3). One qualitative study [69] looked at psychosocial resources instead of focusing on psychosocial risks like most other studies. However, as a single-case study, there are issues with the transferability of the results. The other qualitative study [78] aimed to identify challenges faced by novice community pharmacists at transition to independent practitioners. The three narrative reviews [79,80,81] focused on changing work characteristics in SMEs during the first years of the 21th century. Cooper [80] analyzed the changing world of work and its impact on employees and their families in that period. He focused on new forms of work, affecting most of all employees in SMEs, as well as self-employed and workers outsourced in virtual organizations. Allan et al. [79] investigated challenges of online learning in SMEs. Yuhshy [81] discussed work- and family-related stress as significant problems of SMEs. We identified only three intervention studies [71,73,82]: Casteel et al. [73] evaluated an intervention to reduce violent crimes in SMEs. Magnavita [71] reported an intervention to reduce psychosocial risks at the workplace in SMEs. He described a cost-effective participatory model and emphasized the usefulness of regular health examinations as they can be used to identify problems in the workplace climate and work organization. In a controlled intervention study, Torp [82] reported results of a 2-year training program for managers of small and medium-sized enterprises. He examined the program’s impact on the companies’ health and safety management systems and on the mental and physical health of employees. Applying the SURE-criteria [53], the internal validity of these studies was rated as medium quality, because of some methodological limitations and limited representativeness.

### 3.3. Studies Comparing SMEs and Larger Enterprises

Furthermore, we identified four studies investigating the differences of job stress in SMEs compared to large firms [30,31,63,67]. Whereas Tsai et al. [31] found higher levels of job stress and “higher favorable attitudes toward managers” in SMEs, Lai et al. [63] obtained no firm size effect on overall job stress after adjusting for covariates including individual and organizational characteristics (information on job tenure, contractual status, gender, age, marital status, number of children, caring responsibility, long-term illness, academic qualification, weekly pay, work condition changes, organizational support) in an European (U.K.) sample. Yeh et al. [67] reported higher job demands, higher job insecurity, lower job autonomy and lower career prospect in SMEs, compared to those of large private enterprises and the public sector. Encrenaz et al. [30] investigated the influence of the size of enterprises on mental health and the mediating role of perceived working conditions in this relationship by measuring the outcome “anxious/depressive episodes” in employees. Between micro- enterprises compared to the others, there were differences in perceived working conditions: taking working conditions into account, the risk of “depressive/anxious episodes” was larger in micro-enterprises.

### 3.4. Investigated Psychosocial Demands

As summarized in Table 1, most studies investigated several work characteristics with a focus on factors related to ‘work content and task’ (*n* = 33) and ‘organization of work’ (*n* = 31), followed by factors related to ‘social relations’ (*n* = 30), ‘new forms of work’ (*n* = 8) and the ‘working environment’ (*n* = 7). Only one study considered all five work characteristics to measure work quality in SMEs [29].

A relatively high number of studies assessing the work characteristics ‘work content and task’ included the factors ‘freedom of action’ (*n* = 15), ‘responsibility’ (*n* = 10) and ‘emotional demands’ (*n* = 9). Multiple studies also investigated factors related to ‘organization of work’, mostly looking at ‘work time’ (*n* = 16), ‘work process’ (*n* = 20), and ‘communication/cooperation’ (*n* = 13). However, the correlations of these factors with the outcomes were not always reported. Many studies also investigated the work characteristics ‘social relations’. For example, Berthelsen et al. [85] measured perceived social support in the workplace among Danish dentists, and Agervold et al. [68] measured social contact, social climate and management style in a Danish manufacturing company. Intragroup conflict and social support were assessed by three Japanese studies [62,65,66]. The work characteristics ‘social relations’ in general were measured by four studies [57,60,70,82] and one study referred to Siegrist’s Effort-Reward-Imbalance model (ERI) [71], in which the ERI questionnaire was used to asses occupational stress with items on esteem and job promotion.

The work characteristics ‘working environment’ and ‘new forms of work’ have received less attention. Only three studies measured physiochemical factors: ‘lighting’ was measured by two studies [29,75], ‘climate’ was measured only by Isahak et al. [75]. Rhee et al. [76] assessed the variables ”hazardous work condition” and “handling of hazardous materials”.

Three studies investigated physical factors: “physical occupational activity” [84], “poor physical working conditions” [90] and “heavy physical work, repetitive activities, forced postures and risks of falls” [75]. The factor ‘workplace and information structure’ was only studied by Díaz-Chao et al. [29] (“workspace”). The factor ‘work equipment’ was taken into account by Sonnentag et al. [89] looking at “situational constraints associated with malfunctioning, missing, incomplete, or outdated equipment, tools, or information”. Myers et al. [83] investigated “staff and technical problems”, including ‘equipment breakdown and defective materials’.

The characteristics ‘new forms of work’ was taken into account by eight studies. Job insecurity was measured by five studies [29,63,67,75,91]. Flexible work hours and work-life-balance was taken into account by three studies [29,59,92]. Cooper’s [80] theoretical exploration focused on several aspects of new forms of work, including free-lancers, flexible working hours, and work-family-conflicts related to new forms of work. There was only one study [98] focusing on accessibility and expected availability of workers due to new communication methods. Another study investigated the work-home interference and well-being of self-employed entrepreneurs [59].

### 3.5. Investigated Outcomes and Economic Sectors

Mental health risks and resources were the most commonly studied outcomes. As shown in Table 2, many studies examined mental health outcomes (*n* = 19), with general (work-related) stress outcomes being the second most studied (*n* = 15), followed by outcomes representing business-related outcomes.

General stress and mental health outcomes were assessed more often as risks, business outcomes were assessed almost equally as risks and resources and social relations were examined more often as resources. The only non-self-reported outcomes were blood pressure and the observed rate of violent crimes; all other outcomes were assessed as self-reported outcomes, mostly via questionnaires.

Several studies (*n* = 17) examined manufacturing enterprises (please see Supplementary Table S3). Each of the other economic sectors was considered by less than six studies. Eight studies [30,57,59,63,67,92,93,98] included several sectors; for example; Lai et al. [63] used data of a nationwide survey with participants of all industrial sectors, Godin et al. [59] and Cocker et al. [57] examined entrepreneurs of several sectors. Seven studies [58,79,80,81,86,88,90] did not provide information on particular economic sectors.

## 4. Discussion

In this review, we evaluated the current evidence on work-related psychological stress in SMEs, also summarizing the type of outcomes investigated as well as the economic sectors considered. The majority of the 45 studies were cross-sectional (*n* = 37). We also identified qualitative studies (*n* = 2), narrative reviews (*n* = 3), and intervention studies (*n* = 3). As most studies applied a cross-sectional design investigating relationships between various outcomes and psychosocial factors in the workplace, the number of studies investigating causal relationships was relatively low. Only one study conducted an intervention specifically developed for the SME setting [71] indicating a lack of studies applying a high quality research design (e.g., randomized controlled trials) with a focus on psychosocial stress in the SMEs.

All studies were published from 2004 to 2019 considering very heterogeneous populations of SMEs. We did not find suitable studies from 2000 to 2004. As the definition of SMEs was inconsistent at the beginning of the century, articles from this period may not have met the inclusion criteria of the European definition of SMEs. Since smaller enterprises have become a focus for researchers and policy makers in recent years, this may have resulted in an increased number of publications from 2017 onwards.

Only one study [29] investigated all five dimensions of work-related psychosocial demands as defined in the GDA recommendations [39]. Although these recommendations are based on the European Council Directive [21] and have great similarities with other international classifications (e.g., [12], p. 14), we appreciate that the GDA recommendations have been developed with a focus on the German context and may not be transferable entirely to other settings. Nevertheless, since the Job-Demand-Control Model [36,99] and the extended Job-Demand-Control-Support-(JDCS-) model [100] have been used for decades, work characteristics based on these models, have found their way into many study-designs and work-stress questionnaires. This resulted in a frequent examination of the work pattern ‘work content and task’ although some of the subcategories were hardly considered (‘variability) or not mentioned at all (‘completeness of task’). The work patterns ‘organization of work’ and ‘social relations’ have also been considered frequently. Although the psychosocial health effects of the working environment have been studied as a cause for work-related illness for decades e.g., [101], this work characteristics including ‘physicochemical factors’ or ‘physical factors’ have received little attention from the psychosocial perspective in the studies we identified in this review. In terms of advancing digitalization, topics such as ‘workplace and information structure’ are important fields of research as they involve the risk of information and stimulus satiation [47]. Their psychosocial impact on employees in small and medium-sized enterprises appears to have been poorly researched to date. This may be related to the slow pace of digitization in SMEs requiring financial and human resources, frequently exceeding the means of small enterprises [102]. The European Agency for Safety and Health at Work (EU-OSHA) recognized job insecurity, precarious work, intensification of work and higher requirements for flexibility and mobility from workers as emerging risk for health and safety [2]; nevertheless, ‘new forms of work’ also have received little attention in the setting of SMEs [103].

We also classified the outcomes considered according as defined by the BAUA-project [35] including these subcategories: general (work-related) stress outcomes, health, well-being, factors affecting cardiovascular health, mental health, musculoskeletal system, social relations, and business-related outcomes. Most outcomes were related to the fields of general stress, mental health and business. There appears to be a gap in the investigation of outcomes on physical health, particularly those representing cardiovascular health, even though the link between work-related stress and physical illness is well established [18,19]. Promoting and maintaining the health of employees is the fundamental purpose of occupational medicine. Prevention is therefore particularly important. Work-related resources were, however, less frequently studied than risks, and most outcomes considering resource were related to the fields of ‘social relations’ and ‘business-related outcomes’. Factors considering the prevention of work-related stress and the promotion of a healthy working environment should therefore be considered more frequently in SME-research.

Finally, we categorized the studies identified to the International Standard Industrial Classification of All Economic Activities (ISIC) [51]. With few exceptions, the number of studies allocated to economic sectors corresponded to the frequency with which SMEs in Europe and Asian countries are represented in these sectors [27,104]. In the EU-28, for example, employment was highest in SMEs active in the sectors ‘Construction’ (13,9%) and ‘Wholesale and retail trade; repair of motor vehicles and motorcycles’ (13,6%) between 2016 and 2018 [105]. Although we identified a number of studies (*n* = 9) investigating psychosocial demands in these sectors, the highest number of studies looked at SMEs active in ‘Manufacturing’ (*n* = 17) where the percentage of employment was considerably lower (6.7%). This may be related to differences in lobbying activities which have been shown to be relatively high in the manufacturing sector compared to other sectors including ‘wholesale trade’ [106]. The other economic sectors were partly more typical for large enterprises (e.g., electricity suppliers, oil companies, insurance companies) or public and civil-service institutions (hospitals, schools, public offices) and, as expected, fewer studies were identified here. Within the sector ‘Human health and social work activities’ four studies were identified looking at psychosocial factors in the setting of general dentists’ practices, pharmacies and a welfare and assistance agency for professionals. Although the setting of Myers et al. [83] and Magola [78] was not clearly in the SME area (Myers et al. examined dental practices that were also partly funded by the British National Health Service (NHS), Magola included pharmacists who also worked for larger chains), the studies were included in this review because they represented the sector of healthcare and the organizations under review (dental practices and pharmacies) which ultimately had the structure of an SME. We also expected family or medical practices to be characterized as small businesses, but no corresponding study was identified also indicating that these “enterprises” were unlikely to be categorized as a typical SME [107].

### 4.1. Limitations

This review also has some limitations. Our broad research objective was very useful to give an overview of the current state of research and to detect gaps in knowledge. However, it was not suitable for a “classic” systematic review design and resulted in a heterogeneous sample of included studies and a high number of irrelevant hits, particularly in the database “Business Source Premiere” which identified numerous entries on “financial stress” not related to our research objective. The bias risk has been minimized by a strict application of review methods like systematic literature search and reviewing by independent reviewers. The broad question and the application of the PEO scheme could also be the reason for finding only three intervention studies. It may be appropriate to search particularly for intervention studies in SMEs; however, a recent review from 2019 examined health interventions in SMEs and only one of the included studies met our inclusion criteria [82]. The reasons for this may be related to applying different SME definitions, the inclusion of public institutions or the exclusion of work-related psychological factors as most of the interventions considered in the review [108] dealt with physical fitness or work-safety-interventions.

To provide a more manageable and specific set of results, we have refrained from using ‘family business terms’ in our search string. Apart from using a European definition of SMEs, this could also be a reason why we did not identify studies from South America and Africa. To study psychosocial factors in the family business setting, a new search with a more specific search term would be required. The same may be true for micro-enterprises which may not be covered by our search string in all databases. Furthermore, the consideration of “grey literature” (e.g., governmental reports) may provide further evidence in this context.

Finally, we aimed to categorize all studies identified in this review according to well-established frameworks [35,39,50,51]. Nevertheless, the process of systematization was partly subjective since psychosocial factors are mostly interdependent or interrelated; hence, we could have also chosen different categorizations for some of the studies identified.

### 4.2. Recommendations for Future Research

Psychosocial risks differ between large companies and SMEs [30,31,67]. Since SMEs represent the majority of all companies worldwide, it is important to conduct research specifically focused on smaller enterprises, also including micro-enterprises. As early as 1997, Cooper et al. [109] called for further studies to investigate the long-term effectiveness of stress intervention strategies. With the findings of this review we can renew this demand for the SME setting. In order to offer SMEs effective interventions for the primary prevention of psychosocial risks, the long-term effects of the interventions should be examined applying high-quality study designs. For the development of interventions, it would be desirable to measure all dimensions in which psychosocial risks may occur and resources can be established and consolidated, rather than limiting the assessment of psychosocial stress to single factors. Hereby, the dimensions of psychosocial risks defined by the GDA [39] proofed to be a good framework for the classification of the studies identified. Furthermore, we would like to point out that about one third of the outcomes identified were measured using self-developed items, scales or questionnaire, or by using adapted preexisting questionnaires. On the one hand this complicates the comparison of results with prior research and may impact the validity and reliability of previously established measures. On the other hand, it may be necessary to develop new instruments suitable for a particular research question or setting. Previous research has provided valuable context for the development of new research instruments, also emphasizing the necessity to carefully discuss the pro and cons of using preexisting or newly developed measures e.g., [110].

Working conditions do not only influence the physical but increasingly impact the mental wellbeing of employees [111]. The process of transforming to industry 4.0 with the resulting digitization and emergence of new forms of work (e.g., platform work, remote work, freelancers, home office) has been researched for the last decade and provided valuable insight in central issues or SMEs adapting to the accelerating change of the working environment (e.g., lack of operational capacity for systematic reorganization) [102,112]. Moreover, the trajectory of climate change and the current COVID-19 pandemic have accelerated these processes [113,114]. Particularly the necessity of infection control has resulted in an even greater necessity of work-related mobility and flexibility and is expected to impact the working environment in the long term. As a result, employers and employees are facing new psychosocial risks, e.g., social isolation, increasing technical and social challenges related to electronic communication [115] which has been related to the development of depression, anxiety, self-reported stress, and sleeping disorders [116]. Especially in SMEs, where frequently fewer (financial) resources are available compared to larger companies, the redesign and adaption of a continuously changing working environment is particularly challenging. New evidence of the effectiveness of workplace enhancements in the SME setting could facilitate necessary changes.

## 5. Conclusions

The results of this review highlight that the various psychosocial factors in SMEs have been researched with varying intensity. As Chirico [103] pointed out in 2017, the new work-related risks have not received sufficient attention from the scientific community. This can be recognized here by a lack of studies for the work characteristics “new forms of work” and “working environment”. Within the context of the current COVID-19 pandemic, the relevance of these aspects becomes even more evident. SMEs from the economic sectors “Professional, scientific and technical activities” and “Wholesale and retail trade, repair of motor vehicles and motorcycles” should also be subject to more research, as they appeared to be underrepresented. Due to the lower financial and human resources available in SMEs and a lower awareness of the resulting costs of inadequate health and safety management [12], research for cost-efficient and effective interventions to improve mental health in SMEs is of high relevance to convince entrepreneurs of the benefits of interventions for reducing work-related psychosocial risks.

## Figures and Tables

**Table 1:** Allocation to Dimensions of Psychosocial Factors and Single Work Characteristics According to Beck et al. 2014 [39].

Dimensions and Work Characteristics (Number of Studies)	Work Content and Task (*n* = 33)							Organization of Work (*n* = 31)			Social Relations (*n* = 26)		Working Environment (*n* = 7)				New Forms of Work (*n* = 8)
	Completeness of Task	Freedom of Action	Variability	Information/ Supply of Information	Responsibility	Qualification	Emotional Demands	Work Time	Work Process	Communication/ Cooperation	Colleagues	Managers	Physicochemical Factors	Physical Factors	Workplace and Information Structure	Work Equipment	
Authors, Year																	
**Number of studies examining factor**	**0**	**16**	**3**	**3**	**11**	**7**	**9**	**16**	**21**	**13**	**17**	**19**	**4**	**4**	**1**	**2**	**8**
Myers et al. 2004 [83]						X	X		X							X	
Agervold et al. 2004 [68]							X										
Cooper 2005 [80]																	X
Allan et al. 2005 [79]						X				X							
Bennett et al. 2006 [84]		X							X					X			
Gardner et al. 2006 [74]				X	X	X	X	X		X	X	X					
Nakata et al. 2006 [65]		X							X	X	X	X					
Chuang 2006 [81]			X		X			X	X								
Nakata et al. [66]		X							X	X	X	X					
Tsai et al. 2007 [31]		X				X		X	X			X					
Berthelsen et al. 2008 [85]										X	X						
Casteel et al. 2008 [73]							X										
Rau et al. 2008 [61]		X			X			X	X								
Torp 2008 [82]		X			X						X	X					
Ikeda et al. 2009 [62]		X							X	X	X	X					
Villanueva et al. 2009 [72]												X					
Koskina 2010 [69]							X										
Wang et al. 2009 [86]		X						X		X	X						
Rhee 2010 [76]		X							X	X		X	X	X			
Sawang 2010 [87]												X					
Baillien et al. 2011 [88]							X										
Sonnentag et al. 2012 [89]		X		X				X	X							X	
Nakata 2012 [64]								X									
Cocker et al. 2013 [57]					X			X			X	X					
Kottwitz et al. 2014 [70]									X		X	X					
Lai et al. 2013 [63]		X										X					X
Rahman et al. 2014 [90]					X	X		X	X	X	X	X	X				
Mihic et al. 2015 [60]		X			X			X			X	X					
Magnavita 2015 [91]							X										
Saleem et al. 2016 [92]		X			X			X	X			X					X
Fernet et al. 2016 [58]					X					X	X						
Godin et al. 2017 [59]								X	X		X						X
Lewis et al. 2017 [93]											X						
Isahak et al. 2017 [75]								X	X				X	X			X
Díaz-Chao et al. 2017 [29]		X	X			X		X	X		X	X	X	X	X		X
Hildenbrand et al. 2018 [94]												X					
Magnavita 2018 [71]								X	X			X					X
Sommovigo et al. 2018 [77]							X										
Setti et al. 2018 [95]							X		X								
Magola et al. 2018 [78]					X	X				X	X						
Yeh et al. 2018 [67]		X						X									X
Rastogi et al. 2018 [96]					X				X	X							
Estévez-Mujica et al. 2018 [97]				X					X	X	X						
Encrenaz et al. 2019 [30]		X	X						X			X					
Voss et al. 2019 [98]																	X

**Table 2:** Summary and Classification of Outcomes, Adapted Classification According to Rothe 2017 [35].

Classification	Positive Outcomes/ Resources	[Reference] Applied Measurement Instrument(s)	Negative Outcomes/Risks	[Reference] Applied Measurement Instrument(s)
**General (work-related) stress outcomes**			**Work stress**	[83] The Work Stress Inventory for Dentist (WSID) [74] Self-developed items
			**Work-related stress**	[71] ERI questionnaire, short and validated Italian version [98] Self-developed items
			**Stress reaction**	[76] Self- developed items
			**Workload**	[95] Areas of Work life Survey (Subscale)
			**Work exhaustion**	[96] Oldenburg Burnout Inventory (OLBI) questionnaire (4 items from exhaustion subscale) [92] Pre-used questionnaire
			**Employees’ experience of overall job stress**	[63] Constructed scale of Workplace Employment Relations Study (WERS) 2011
			**Level of stressful situations at work**	[74] Self-developed items
			**Stressful work-related conditions**	[83] Work Stress Inventory for Dentists (WSID), adapted
			**Perceived stress**	[83] Perceived Stress Scale (PSS) [76] Self-developed item
			**Personal stress**	[74] Self-developed items
			**Psychological stress**	[68] The Psychosocial Work environment and Stress Questionnaire (PWSQ)
			**Psychological distress**	[57] Kessler (K10) Screening Scale for Psychological Distress [86] The Taiwanese Depression Questionnaire (TDQ)
			**Psychological pressure**	[60] self-developed items
			**Job complexity**	[96] pre-used items
			**Work-home interference**	[59] Kelloway’s work-family conflict questionnaire
**Health**	**Health**	[83] The 12-item General Health Questionnaire (GHQ-12) [83] health-related behaviors questionnaire; minor ailments and symptoms checklist (unspecified)	**Self-reported sick-leave**	[68] The Psychosocial Work environment and Stress Questionnaire (PWSQ)
	**Self-rated health (SRH)**	[64] Self-developed item [59] Self-developed item	**Occupational injury**	[65] Self- developed single item
**Well-being**	**Quality of life**	[75] The WHO quality of life assessment instrument (WHOQOL-Bref)	**Vital exhaustion**	[61] Maastricht Questionnaire (MQ)
**Factors affecting cardiovascular health**	**Leisure time physical activity**	[84] Modified version of a pre- used semi-quantitative activity questionnaire	**Metabolic syndrome component**	[91] Common diagnosis criteria
			**Sleep-related breathing disturbance**	[66] Pre-used adopted single item
			**Increased blood pressure ^1^**	[61,70] 24h-automatically-recorded blood pressure
**Mental health**	**General psychological health**	[95] General Health Questionnaire (GHQ-12)	**Psychosomatic symptoms**	[68] The Psychosomatic Work Environment and Stress Questionnaire (PWSQ)
	**Emotion management**	[69] Qualitative methods (semi-structured interviews, non-participant observations)	**Fatigue**	[86] Chinese version of Checklist individual Strength (CIS)
	**Mental wellbeing (absence of anxiety and depression symptoms)**	[71] Goldberg Anxiety and Depression scale (GADS)	**Mental fatigue**	[68] The Psychosomatic Work Environment and Stress Questionnaire (PWSQ)
	**Resilience**	[96] Pre-used items	**Burnout**	[94,97] OLBI questionnaire [58] French version of the Burnout Measure, Short version (BMS) [67] Chinese version of Copenhagen Burnout Inventory (C-BI)
	**Coping self-efficacy**	[77,95] Seven-item Coping Self-Efficacy scale (CSE-7)	**Depressive symptoms**	[62] Japanese version of the Center for Epidemiologic Studies Depressive Symptoms Scale (CES-D)
			**Depressive Episodes**	[30] Hospital Anxiety and Depression Scale (HADS-D)
			**Depression**	[61] Hospital Anxiety and Depression Scale (HADS-D) [74] self-developed items
			**Anxious Episodes**	[30] Hospital Anxiety and Depression Scale (HADS-A)
			**Anxiety**	[61] Hospital Anxiety and Depression Scale (HADS-A)
			**Sleep disorders**	[61] Schlaf-Wach-Erlebnisliste [Sleep Wake Experience List] (SWEL)
			**Post-traumatic stress symptoms**	[95] The six-item Impact of Event-Revised scale (IES-R)
			**Post-traumatic Stress Disorder (PTSD)**	[77] The six-item Impact of Event-Revised Scale (IES-R)
			**Psychological strain**	[87] 2 items of the General Health Questionnaire (GHQ)
	**Job satisfaction**	[95] Pre-used single item [76] Self-developed item [87] Short version of Minnesota Satisfaction Questionnaire (MSQ)	**Job dissatisfaction**	[83] The job dissatisfaction Measure
**Musculoskeletal system**			**Musculoskeletal pain**	[82] Health Complains Questionnaire
**Social relations**	**Social support Seeking**	[95] Coping Orientation to Problem Experienced scale (COPE-IV)	**Bullying at work**	[93] Self-developed items [68] 12-item checklist partly based on the Negative Acts Questionnaire (NAQ) [88] Negative Acts Questionnaire (NAQ)
	**Sources of Support**	[74] Self-developed scales	**Harassment**	[93] Self-developed items
	**Perceived practical support**	[85] Self-developed scales	**Observed number of violent crimes ^1^**	[73] Crimes, identified by L.A. Police departments
	**Social support**	[82] Self-developed items [74] Self-developed scales		
	**Emotional support**	[85] Self-developed scales		
	**Availability of contact with colleagues**	[85] Self-developed scales		
	**Management support**	[82] Self-developed items		
**Business-related outcomes**	**Success in a family firm**	[60] Self-developed items	**Presentisms**	[57] Self-developed item
			**Absenteeism**	[57] One item from the WHO Health and Work Performance Questionnaires (HPQ)
	**Job quality**	[29] Scales of 2008 and 2011 Quality of life survey (ECVT in Spanish)	**Disengagement**	[96] Five items of the OLBI questionnaire
	**Performance enhancement**	[90] Unspecified questionnaire (probably self-developed)	**Productivity loss**	[57] Self-developed item
	**Promotion opportunities**	[31] Self-developed items based on WERS, other pre-used questions	**Turnover intention**	[92] Pre-used questionnaire
	**Job security**	[31] Self-developed Questionnaire based on WERS, other pre-used questions	**Intention to leave**	[31] Self-developed items based on WERS, other pre-used questions [72] Five-item scale by Wayne et al. (see Reference)
	**Proactive work behavior**	[89] Seven-item scale by Frese et al. (see Reference)	**Training needs**	[90] Unspecified questionnaire (probably self-developed)
	**Importance of work**	[59] Mow’s question on the centrality of work		

^1^—non-self-reported outcomes.

## Supplementary Materials

The following are available online at https://www.mdpi.com/1660-4601/17/20/7446/s1, Table S1: Search Strategy for Pubmed (NCBI), Table S2: Summary of study characteristics, Table S3: Allocation to economic sectors according to the International Standard Industrial Classification of All Economic Activities (ISIC).
