# Supplementary material for: Work-Related Psychosocial Stress in Small and Medium-Sized Enterprises: An Integrative Review
Source: Int J Environ Res Public Health. 2020 Oct 13;17(20):7446. doi: 10.3390/ijerph17207446 (PMC7650689; doi:10.3390/ijerph17207446)
Supplement: Supplementary file 1 [file ijerph-17-07446-s001.zip › Supplementary table S2 - Summary of study characteristics_2.pdf]

Supplementary table S2. Summary of study characteristics.

**Authors and year; country**	**Year of data collection**	**Topic**	**Design**	**Data-collecting methods**	**Type of enterprise; sample size (*n*); industrial classification according to ISIC Rev. 4 [51]**	**Investigated psychosocial demands (according to GDA [39])**	**Outcomes**	**Data collection instrument(s) ***
Myers et al. 2004 [83]; United Kingdom	1999-2000	Investigation of overall stress, work stress and health in general dental practitioners (GDPs).	Cross-sectional study	A nationwide survey was conducted on GDPs of health authority lists.	General private dental practices; *n* = 2441 GDPs; Q. Human health and social work activities	1. Work content and task: qualification, emotional demands<br>2. Organization of work: work process<br>3. Social relations: -<br>4. Working environment: work equipment<br>5. New forms of work: -<br>(subsequently, we only report work characteristics investigated)	Main outcomes: perceived stress; health<br><br>Minor outcomes: work environment: (stressful work-related conditions; job dissatisfaction)	Perceived stress[1]: perceived stress scale<br>Health[1]: General Health Questionnaire (GHQ-12) Health-related behaviors questionnaire (unspecified), minor ailments and symptoms checklist (unspecified)<br>Work environment[1,2]: specific dental questions (self-developed), The Work Stress Inventory for Dentists (WSID), adapted by the authors<br>Job dissatisfaction[1]:

								the Job Dissatisfaction Measure
Agervold et al. 2004 [74]; Denmark	Un-known	The stress levels of bullied and non-bullied employees were assessed and the relationship between bullying and other psychosocial factors was investigated.	Cross-sectional study	The study was part of a survey to map quality of work environment and employee well-being in one company.	Food manufacturing company; *n* = 202 employees; C. Manufacturing	1. Work content and task: emotional demands<br>3. Social relations: colleagues, managers	Main outcome: bullying at work<br><br>Minor outcomes: mental fatigue; self-reported sick-leave; psychological stress; psychosomatic symptoms	<u>Bullying at work[1]:</u> checklist of negative acts, partly based on the Negative Acts Questionnaire' (NAQ); additionally, a single item was used<br><u>Working conditions[2], Minor outcomes[1]:</u><br>'The Psychosocial Work Environment and Stress Questionnaire' (PWSQ)
Cooper 2005 [80]; country not reported	No data collected	Theoretical discussion of the future of work in general with a focus on career,	Narrative review	Unsystematic literature review	SMEs, freelancer; *n* = not relevant; no sector reported	5. New forms of work	-	-

		stress and well-being of small and medium-sized enterprises (SMEs).						
Allan et al. 2005 [79]; country not reported	No data collected	The stress caused by online collaboration in SME staff was researched. Online team roles as possible stressors were reported.	Narrative review	Unsystematic literature review	SME; *n* = not relevant; no sector reported	1. Work content and task: qualification<br>2. Organization of work: communication/cooperation<br>3. Social relations: managers<br>5. New forms of work	-	-
Bennett et al. 2006 [84]; USA	Unknown	The relationship between occupational stress and LTPA (leisure time physical activity) and their modification by race or ethnicity was investigated.	Cross-sectional study	The study was based on data of a randomized controlled trial of the Healthy Directions small business study. Data were collected by interviewer-administered survey	26 SMEs: 50-150 employees<br>*n* = 1442;<br>C. Manufacturing	1. Work content and task: freedom of action<br>2. Organization of work: work process<br>4. Working environment: physical factors	Leisure time physical activity (LTPA)	LTPA[1]:<br>a semi-quantitative activity Questionnaire<br>job strain[2]:<br>A modified version of Karasek's Job Content Questionnaire (JCQ)
Gardner et al. 2006 [60]; New Zealand	1999	The causes of work-related stress in the veterinary field were explored, as well as what stress levels were	Cross-sectional study	A postal survey was distributed to veterinarians registered with the Veterinary Council of New Zealand.	Veterinarians;<br>*n* =849;<br>M. Professional, scientific and technical activities	1. Work content and task: information/ supply of information; responsibility; qualification; emotional demands<br>2. Organization of work: working time; communication/	Work stress;<br>level of stressful situations at work;<br>personal stress;<br>sources of support;<br>social support;<br>depression	All measurements[1,2]:<br>survey, developed by the Veterinary Council of New Zealand and New Zealand Veterinary Association

		experienced and what social support veterinarians used to cope with work-related stress.				cooperation;<br>3. Social relations: colleagues; managers		
Nakata et al. 2006 [63]; Japan	2002	The association between the broad aspects of job stress and occupational injuries in SMEs was examined.	Cross-sectional study	Factories randomly selected from a directory were contacted by phone and asked to participate in a self-administered questionnaire survey.	244 SMEs;<br>n = 1770;<br>C. Manufacturing	1. Work content and task: freedom of action; qualification<br>2. Organization of work: work process; communication/ cooperation<br>3. Social relations: colleagues; managers	Occupational injury	Occupational injury[1]: dichotomized single question item<br>Job stress[2]: The Japanese version of the Generic Job Stress Questionnaire (GJSQ), developed by the US National Institute for Occupational Safety and Health.
Chuang 2006 [81]; country not reported	No data collected	Stress-related factors in small businesses were examined. (role conflict, work family conflict, role stress, levels of job satisfaction, role ambiguity, role overload)	Narrative review	Unsystematic literature review	Small businesses (50-200 employees) were theoretically discussed;<br>no sector reported	1. Work content and task: variability; responsibility<br>2. Organization of work: working time; work process	-	-

Nakata et al. 2007 [64]; Japan	2002	The association of job stress with sleep-related breathing disturbance (SBD) was investigated.	Cross-sectional study	Factories randomly selected from a directory were contacted by phone and asked to participate in a self- administered questionnaire survey.	292 SMEs; 1–158 workers; n = 1940 males; C. Manufacturing	1. Work content and task: freedom of action; qualification<br>2. Organization of work: work process; communication/ cooperation;<br>3. Social relations: colleagues; managers	sleep-related breathing disturbance (SBD)	SBD[1]: adopted single-question item, used before in several studies<br>perceived job stress[2]: The Japanese version of the Generic Job Stress Questionnaire (GJSQ)
Tsai et al. 2007 [31]; United Kingdom	2005	The association of firm size with job satisfaction was investigated.	cross-sectional study	Interviews with managers to assess context information and a structured employee questionnaire were used.	SMEs, 2-250 employees; n = 384; C. Manufacturing, J. Information and communication, R. Arts, entertainment and recreation	1. Work content and task: freedom of action; qualification<br>2. Organization of work: working time; work process<br>3. Social relations: managers<br>5. New forms of work	dependent variables: job autonomy; attitudes to managers; extent of and satisfaction with training; promotion opportunities; job security; intention to leave; work pressure and effort;	dependent variables[1]: self-developed, pilot-tested, structured employee questionnaire, based on the WERS questions (Kersley et al., 2006) and those reported by Gallie et al. (2004) (a series of standard questions deployed in British surveys since 1986) also new questions were designed.<br>market strength[2]: self-developed index (from the information

								provided by managers and expert opinions) <u>formality[2]:</u> overall index of the formality of HRM practices, followed by Way (2002).
Berthelsen et al. 2008 [85]; Denmark	2002	The extent to which Danish general dental practitioners perceived support from colleagues and the relation of this support to demographic and work-related background was investigated.	Cross-sectional study	A postal questionnaire was used to collect data of a random sample of dentists.	Dentists from the register of the Danish Dental Association *n* = 221; Q. Human health and social work activities	2. Organization of work: communication/ cooperation 3. Social relations: colleagues	Perceived practical support; availability of contact with colleagues; emotional support	All variables[1]: self-developed and pilot-tested questionnaire
Casteel et al. 2008 [59]; USA	1997-2000	The effectiveness of a robbery and violence prevention program in small businesses in Los Angeles was introduced.	Intervention study	Basic data as well as follow-up data on risk factors for violence and preventive measures were collected by security consultants. Violent crimes were identified by linking data from	Small, independently owned businesses; 305 intervention businesses, 96 control businesses *n* = ?; G. Wholesale and retail trade; repair of motor vehicles	1. Work content and task: emotional demands	Number of violent crimes (after a consultation on violence-preventing measures such as organizational and environmental improvements, compared to a control group without	Number of crimes identified by Los Angeles Police Department records

				an electronic police directory with the address data of the companies participating in the study.	and motorcycles; I. Accommodation and food service activities		intervention)	
Rau et al. 2008 [66]; Tri-border corner of Germany, Czech Republic and Poland	2004	Work characteristics and success of entrepreneurs and the link to the health of entrepreneurs were researched.	Cross-sectional study	Data on businesses and work characteristics were collected by structured interviews and by questionnaires. On a working day, participants received 24-hour outpatient blood pressure monitoring.	Entrepreneurs; 5-50 employees; *n* = 53; A. Agriculture, forestry and fishing J. Information and communication	1. Work content and task: freedom of action; responsibility 2. Organization of work: working time; work process	Depression; anxiety; vital exhaustion; sleep disorders; increased blood pressure	Anxiety and depression[1]: Hospital Anxiety and Depression Scale (HADS-D) Vital exhaustion[1]: Maastricht Questionnaire (MQ) Sleep disorder[1]: Schlaf-Wach-Erlebensliste [Sleep Wake Experience List] (SWEL) Blood pressure[1]: 24h - blood pressure monitoring Working conditions[2]: Job Demand/ Control-Questionnaire (JDC)

Torp 2008 [82]; Norway	Unknown	A two-year training program in health and safety management (H&S) for managers of motor vehicle repair garages was examined with regard to implementation of H&S management procedures and effects on workers' perception on the physical and psychosocial working environment and their health.	Intervention study	The effects were investigated by questionnaires sent to managers (questionnaire on H&S) and workers (questionnaire on the working environment and musculoskeletal pain) before and after a HS-training intervention.	Motor vehicle repair garages; 2-140 employees; (*n* = 226);<br>G. Wholesale and retail trade; repair of motor vehicles and motorcycles	1. Work content and task: freedom of action; responsibility<br>3. Social relations: colleagues; managers	Implementation of H&S management procedures:<br><br>psychosocial working environment: decision authority;<br><br>social support; management support<br><br>health measure: musculoskeletal pain	Management procedures and psychosocial working environment[1,2]: self-constructed questionnaire<br><br>Musculoskeletal pain[1]: Health Complaints Questionnaire
Ikeda et al 2009 [61]; Japan	2002	Factors associated with depressive symptoms were investigated in SMEs.	Cross-sectional study	Survey with a self-rating questionnaire.	292 SMEs; 1–158 employees;<br>*n* = 2302;<br>C. Manufacturing	1. Work content and task: freedom of action; qualification<br>2. Organization of work: work process; communication/ cooperation<br>3. Social relations: colleagues; managers	Depressive symptoms	Depressive symptoms: Japanese version of the Center for Epidemiologic Studies Depressive Symptoms Scale (CES-D)<br>Job stressors: Japanese version of

								Generic Job Stress Questionnaire (GJSQ)<br>SME stress questions (SMESQ)
Villanueva et al. 2009 [58]; Australia	Unknown	SME employees´ intentions to leave the organization were examined in relation to their perceived occupational stress.	Cross-sectional study	After information in a staff meeting a questionnaire was distributed to the employees by the management. The completed questionnaire was returned to the researchers by letter.	SMEs with 50-100 employees;<br>*n* = 154;<br>C. Manufacturing,<br>M. Professional, scientific and technical activities<br>N. Administrative and support service activities	1. Work content and task: responsibility<br>2. Organization of work: working time<br>3. Social relations: colleagues; managers	Intention to leave	<u>Intention to leave:</u> five-item scale developed by Wayne et al. (1997).<br><u>Perceived organizational support:</u> the short version of the Survey of Perceived Organizational Support (SPOS)<br><u>Occupational stress</u> <u>:</u><br>"sources of pressure" scale<br><u>Job satisfaction:</u> 12-item scale, based on the Job Descriptive Index, developed by Smith, Kendall, and Hulin (1969)
Koskina 2010 [75]; Greek	Unknown	By looking at the context and control of emotional performances and the work conditions that	Qualitative case study	Data was obtained through interviews with customer service representatives, supported by interviews with	SME<br>*n* = 28;<br>J. Information and communication	1. Work content and task: emotional demands	Emotion management	Semi-structured interviews, supplemented by limited non-participant observation

		would create a positive working atmosphere, the nature of emotion management in call-centers was explored.		different levels of management and supplemented by limited non-participant observations.				
Wang et al. 2009 [86]; Taiwan	2005-2006	The relationship between psychosocial job characteristics and fatigue was investigated, and risk factors for fatigue among employees of SMEs enterprises were determined.	Cross-sectional study	During a health examination a blood sample and self-administered questionnaires on demographic information, work and lifestyle as well as psychosocial work characteristics, fatigue and mental stress were collected from each participant.	Small enterprises < 50 employees; *n* = 647; no sector reported	3. Social relations: managers	Fatigue	Fatigue[1]: Chinese version of checklist individual strength (CIS)<br>Psychosocial work characteristics[2]: validated Chinese version of the Job Content Questionnaire (JCQ)<br>Psychological distress[2]: The Taiwanese depression questionnaire (TDQ)
Rhee 2010 [67]; Korea and Japan	2001	The effect of trust on work stress was investigated. Three dimensions of trust were examined with regard to their	Cross-sectional study	Data was collected by self-administered and structured questionnaires of a randomly selected sample of Korean and Japanese SME	Small and medium-sized firms < 120 employees; Korean workers *n* = 376 Japanese workers *n* = 77; C. Manufacturing	1. Work content and task: freedom of action<br>2. Organization of work: work process communication/ cooperation;<br>3. Social relations: colleagues; managers<br>5. 4. Working	Main outcome: perceived stress;<br><br>Secondary outcomes: stress reaction: stress symptom prevalence; job satisfaction	Self-developed questionnaire[1,2]:<br>Perceived stress[1]: single question item<br>Stress reaction[1]: two dimensions: stress symptom prevalence

		different positions as antecedent, moderating and mediating factors in the cause of stress.		workers.		environment: physicochemical factors		(measured by summation of subjective severity of non-specific stress symptoms) and job satisfaction (single item) Work-related stressors[2]: scales, selected from the NIOSH Generic Job Stress Questionnaire Workers trust[2]: modified version of the Organizational Trust Inventory, Yamagishi's Scale of Trust
Sawang 2010 [87]; Thailand	Unknown	The mediating and the moderating effect of perceived managerial support on role stressors and psychological outcomes were tested.	Cross-sectional study	After contacting entrepreneurs by letter and/or telephone and after permission to collect data, questionnaires were posted to organizations with pre-paid return envelops.	13 small manufacturing firms *n* = 380; C. Manufacturing	3. Social relations: managers	Psychological strain; job satisfaction	Psychological strain[1]: 12 items of the General Health Questionnaire (GHQ) Job satisfaction[1]: Minnesota Satisfaction Questionnaire (MSQ) short version by Weiss et al. (1967) perceived managerial

								support[2]:<br>short version of the Survey of Perceived Organizational Support (SPOS) by Rhoades and Eisenberger (2002)<br>role stressors[2]:<br>The 14-item scale measures role ambiguity and role conflict (Rizzo et al. 1970).
Baillien et al. 2011 [88]; Belgium	2005	Organizational correlates of workplace bullying in SMEs were examined.	Cross-sectional study	Questionnaires were sent to businesses after contacting the organization's manager and asked for their cooperation.	39 SMEs <100 employees<br>*n* = 358;<br>no sector reported	1. Work content and task: emotional demands<br>3. Social relations: colleagues; managers	Bullying at work	Bullying at work[1]:<br>Negative Acts Questionnaire (NAQ)<br>Organizational characteristics[2]:<br>Self-developed questionnaire with four validated scales
Sonnentag et al. 2012 [89]; Germany	Unknown	It was examined whether role breadth self-efficacy is the underlying mechanism of the positive relationship of job stressors and job control with proactive	Cross-sectional study	After having received the CEOs' consent, paper-and-pencil questionnaires were sent to the participants (one each for the participant and the direct supervisor).	Small and medium-sized companies;<br>*n* = 140;<br>C. Manufacturing	1. Work content and task: freedom of action; information/supply of information<br>2. Organization of work: working time; work process<br>4. Working environment: work equipment	Proactive work behavior	Proactive behavior[1]:<br>supervisor-rated proactive behavior and personal initiative with the seven-item scale by Frese et al. (1997).<br>Taking charge[2]:<br>six items from the measure developed by Morrison and

		behavior, and whether supervisor-rated proactive behavior is as positively related to job stressors as proactive behavior itself.						Phelps (1999)<br><u>Role breadth self-efficacy[2]:</u><br>nine items from the measure developed by Parker (1998)<br><u>Work characteristics[2]:</u><br>(job control and job stressors) self-report scales developed by Semmer, Zapf, and Dunckel (1999),
Nakata 2012 [62]; Japan	2002	The associations between work hours, sleep status and self-reported health, among full-time employees, was investigated.	Cross-sectional study	After agreement of the companies, questionnaires were distributed to all employees during site visits.	296 SMEs; 1-158 employees; *n* = 2884; C. Manufacturing	1. Work content and task: freedom of action; qualification<br>2. Organization of work: work process; communication/ cooperation<br>3. Social relations: colleagues; managers	Self-rated health (SRH)	<u>SRH[1]:</u><br>one question: How would you describe your health during the past 1-year period? (very good / good / poor / very poor)<br><u>Daily sleep hours and subjective sleep sufficiency[2]:</u><br>two self-developed items<br><u>Work hours[2]:</u><br>assessed by an open-ended question (self-developed)
Cocker et al. 2013 [57];	Unknown	The prevalence of high/very	Cross-sectional	Baseline data from the	Managers of SMEs < 200	1. Work content and task: Responsibility;	Main outcomes: absenteeism;	<u>Interview survey, not specified[1,2]</u>

Australia	high psychological distress, past-month sickness absenteeism and presentism days in SME owner/managers were investigated. In addition the associated, self-reported lost productivity and the associations between work, non-work and business-specific factors and work attendance behaviors were examined.	study	evaluation of the Business in Mind program, a mental health promotion intervention amongst SME owner/managers were used. The method of data collection is not described, but questionnaires seem to have been used.	employees<br>*n* = 217;<br>several industrial sectors	2. Organization of work: working time;<br>3. Social relations (not further differentiated)	presentism;<br>Conscientiousness;<br>productivity loss (the percentage of time SME owner/managers thought they were as productive as usual<br>when they continued to work during illness)<br><br>Minor outcome: psychological distress;	<u>absenteeism days[1]:</u><br>one item from the World Health Organizations Health and Work Performance Questionnaires (HPQ)<br><u>Presentism[1]:</u><br>item: "How many days in the last 4 weeks did you got to work while suffering from health problems?"<br><u>Lost productive time[1]</u>:<br>item: "On these days, when you went to work suffering from health problems, what percentage of your time were you as productive as usual?"<br><u>Conscientiousness[1]:</u><br>five-item measure from the NEO Personality Inventory-Revised (NEO-PI-R)<br><u>Psychological distress[2]:</u>

Kessler (K10) Screening Scale for Psychological Distress

Health related factors[2]:

general self-rated health was assessed by the first item of the SF-12, *Treatment* was measured by participant responses regarding the receipt of professional medical help for a mental health concern in the three months prior to the survey.

Work related well-being factors[2]:

Business confidence was measured by one-item measure; *Job satisfaction* was assessed by a 3–item measure; *Work/life balance* was determined by a 4-item measure.

Business characteristics[2]: one

								item each for *number of employees supervised, number of employees in organization, work hours*
Kottwitz et al. 2014 [76]; Switzerland	Unknown	The prevalence of work-related stressors and the association with blood pressure were investigated across one week of intense work.	Cross-sectional study	Work characteristics were assessed by questionnaire at the beginning of the study, after that the employees participated in repeated outpatient blood pressure measurements over one week.	Complete staff of a small enterprise *n* = 7; J. Information and communication	2. Organization of work: work process; 3 Social relations (not further differentiated)	Increased blood pressure	24h-automatically recorded blood pressure[1]<br><br>Time pressure[2]: a short self-report version of the Instrument for Stress Oriented Task Analysis<br>Social stressors[2]: 8-item scale developed by Frese and Zapf
Lai et al. 2015 [72]; United Kingdom	2011-2012	Employees' experience of work-stress was examined in relation to firm size.	Cross-sectional study	A matched dataset of the Workplace Employment Relations Survey 2011, a national work-place survey in United Kingdom, was used.	470 SMEs *n* = 2250; several industrial sectors	1. Work content and task: freedom of action; 3. Social relations: Managers; 5. New forms of work	Employees' experience of overall job stress	Employees' experience of overall job stress[1]: constructed scale of six items of Workplace Employment Relations Survey 2011 (WERS2011)<br>Job stressors[1]: scales were developed from WERS 2011,

								adopted to the “An Organizational Stress Screening Tool” (ASSET) Questionnaire
Rahman et al. 2014 [90]; Malaysia	Unknown	The factors of the relationship between performance enhancement and developing training needs of SME-employees and occupational stress were identified.	Cross-sectional study	An unspecified pilot-tested questionnaire was used. It was not clearly described if the questionnaire was self-developed or how it was distributed.	SMEs in Melaka; *n* = 180; no sector reported	1. Work content and task: responsibility; qualification; 2. Organization of work: working time; work process; communication/cooperation; 3. Social relations: colleagues; managers; 4. Working environment: physical factors	Training needs; performance enhancement	Self-administered, pilot tested questionnaire, unspecified[1,2]
Mihic et al. 2015 [71]; Serbia	2012	Owners’ perception of stress, caused by flexible working times and high levels of responsibility and the influence of family member employees on the entrepreneurial success was investigated.	Cross-sectional study	A self-developed questionnaire was used in a nationwide survey of owners of family-owned businesses.	Micro-enter-prises and SMEs; *n* = 2206; C. Manufacturing; S. Other service activities (not specified)	1. Work content and task: freedom of action; responsibility; 2. Organization of work: working time; 3 Social relations (not further differentiated)	Psychological pressure; success in a family firm;	Self-developed survey, containing 22 questions, generated from a theoretical research of the field of study[1,2]

Magnavita 2015 [91]; Italy	Unknown	The association between psychological injury and metabolic syndrome (MES) was evaluated.	Cross-sectional study	As part of their routine medical examination at the workplace, employees completed a self-developed questionnaire containing the Psychological Injury Risk Indicator (PIRI) questionnaire. In addition, biological data were determined and a blood sample was taken.	20 small companies; *n* = 571<br>F. Construction<br>G. Wholesale and retail trade repair of motor vehicles and motorcycles<br>Q. Human health and social work activities	1. Work content and task: emotional demands	One metabolic syndrome component	Metabolic syndrome components[1]:<br>• central obesity (defined as BMI>30 kg/m2, or increased waist circumference with ethnicity-specific values)<br>• elevated triglyceride (TGs) level: > 150 mg/dL (1.7 mmol/L) or specific treatment for this lipid abnormality<br>• reduced HDL cholesterol (HDL-c):<br>• < 40 mg/dL (1.03 mmol/L) in males, < 50 mg/dL (1.29 mmol/L) in females, or specific treatment for this lipid abnormality<br>• high blood pressure (BP): systolic BP > 130 or diastolic BP<br>• >85 mm Hg, or treatment of previously

								diagnosed hypertension<br>• high fasting plasma glucose (FPG): >100 mg/dL (5.6 mmol/L), or previously diagnosed type 2 diabetes<br><br><u>Psychological injury[2]:</u> questionnaire, including scales of the Psychological Injury Risk Indicator (PIRI), which was developed for the early detection of emerging psychological injuries among workers
Saleem et al. 2016 [92]; Pakistan	Unknown	To address employees' turnover among small and medium firms in Pakistan's service sector, the antecedents of work exhaustion and	Cross-sectional study	A self-rated questionnaire was used to collect data for independent variables on the start of a bus trip from the employees of the bus companies,	5 bus services; 15-86 employees; *n* = 281 several industrial sectors	1. Work content and task: freedom of action; responsibility;<br>2. Organization of work: working time; work process;<br>3. Social relations: Managers;<br>5. New forms of work	Dependent variable: turnover intention<br><br>Mediating variable: work exhaustion	Questionnaire, reported and validatetd by Sondhi et al. (2008) and Ahuja et al. (2007) (contains scales developed by Moore(2000) and Beehr (1976))[1,2]

		the missing linchpin were explored.		the same participants were asked to fill in the survey on mediator and dependent variables on the bus return.				
Fernet et al. 2016 [69]; France	Unknown	The individual and contextual factors, that make small-to-medium enterprise owner-managers vulnerable for burnout, were investigated to gain a deeper understanding of their occupational stress.	Cross-sectional study	The multi-wave study collected data at four different times (T1-T4) over an eight-month period using a short phone questionnaire. Sociodemographic data and entrepreneurial orientation were initially collected (T1). Scales designed to assess job stressors related to SME management (T2), loneliness (T3), and burnout (T4) were asked afterwards.	Managers of SMEs, 3-250 employees<br>n = 377;<br>no sector reported	1. Work content and task: Responsibility;<br>2. Organization of work: communication/ cooperation;<br>3. Social relations: colleagues	Burnout	Burnout[1]:<br>The French version of the Burnout Measure, Short Version (BMS)<br><br>Job stressors related to SME management[2]:<br>five items developed by Torres and Lechat (2012)<br>Entrepreneurial orientation[2]:<br>the 9-item scale developed by Covin and Slevin (1989)<br>Occupational loneliness[2]:<br>single item

Godin et al. 2017 [70]; Belgium	2012-2015; 2015-2016	The working conditions of entrepreneurs in very small companies were assessed. The impact of these conditions on their health and well-being as well as the interference with their private lives was investigated.	Cross-sectional study	One study used a combination of in-depth interviews, self-administered questionnaires, and on-site observation methodology, the other study included a survey made available online through the Brussels Enterprise Agency, Impulse, Brussels.	Entrepreneurs of SMEs (80% < 5 employees); compared to 104 small retailers *n* = 140/ *n* = 104; several industrial sectors	2. Organization of work: working time; work process; 3. Social relations: Colleagues; 5. New forms of work	Self-reported health ; well-being (self-reported); work-home interference importance of work	2012-2015: in-depth interviews, unspecified questionnaires, and on-site observation[1,2] 2015–2016: survey, in both French and Dutch versions with multiple health indicators (self-reported)[1,2] Subjective health status[1]: a close-ended question in five categories clubbed into two groups ("good/very good" and "average/(very) bad") Work-home interference[1]: Kelloway's questionnaire Importance of work[1]: Mow's question on the centrality of work

Isahak et al. 2017 [65]; Malaysia, Indonesia, Thailand, Vietnam	2014-2015	The study examined Quality of Life (QOL) among SME-workers in four Asian countries and the association of Workplace Exposures to the physical, psychological, social and environmental domain of QOL.	Cross-sectional study	An anonymous self-administered and self-designed questionnaire was distributed to workers and collected by researchers a few days later.	SMEs in Jakarta, Kuala Lumpur, Bangkok and Can Tho; *n* = 2430, 500 from each country C. Manufacturing	2. Organization of work: working time; 4. Working environment: physiochemical factors; physical factors; 5. New forms of work	Quality of life (QOL) (physical domain, psychological domain, social domain, environmental domain)	Quality of life[1]: The WHO quality of life assessment instrument (WHOQOL-Bref) Chronic fatigue[1]: four questions proposed by Albert et al.'s chronic fatigue questionnaire Sociodemographic factors and work characteristics; work environment and ergonomic conditions[2]: assessed in a self-designed survey
Lewis et al. 2017 [93]; United Kingdom	2008	By using data from a national survey, the study examined the relationship between work-related stressors and bullying and harassment in British SMEs.	Cross-sectional study	Data of a secondary self-completed survey of "The Fair Treatment at Work Survey" were used. After cleaning data, 1357 fully completed questionnaires for analysis in the SME category remained.	SMEs (<250 employees) *n* = 1357 several industrial sectors	1. Work content and task: emotional demands; 3. Social relations: colleagues; managers	Bullying at work; harassment	Secondary self-completion survey of 'The Fair Treatment at Work Survey' (two items asked whether respondents were subject to bullying and harassment at work[1] and 31 items represented work-related stressor influences[2], originated from the

								HSE's 'Management Standards' (2008))
Díaz-Chao et al. 2017 [29]; Spain	2008 – 2010	Analysis of the extent to which a set of related job quality dimensions, by enterprise-size class, have evolved between 2008 and 2010. Results were related to the recession.	Cross-sectional study	Data collection for Quality of Working Life Survey for 2008 and 2010 was done by means of computer-assisted telephone interviews or, if not possible by personal interviews.	SMEs< 250 employees (micro (n = 1-10) small (n = 11-50) medium (n = 51-249) Sample: 2008: n = 3138 2010: n = 2917 A. Agriculture, forestry, fishing C. Manufacturing F. Construction Service (not specified))	1. Work content and task: freedom of action; variability; qualification; 2. Organization of work: working time; work process; 3. Social relations: colleagues; managers; 4. Working environment: physicochemical factors; workplace and information structure; 5. New forms of work	Job quality	Quality of Working Life Survey (ECVT, as abbreviated in Spanish)[1,2] for 2008 and 2010 (Statistical survey conducted by Spain's Ministry of Employment and Social Security)
Hildenbrand et al. 2018 [94]; Germany	Unknown	The correlation of transformational leadership to employee burnout was investigated by considering thriving at work (personal resource) and employees' openness to experience (a	Cross-sectional study	A questionnaire as paper-and-pencil- or online version containing previously translated and validated scales (except for thriving) was used. The items pertaining to thriving were translated using back-translating	One midsized company; n = 148 C. Manufacturing	3. Social relations: managers	Burnout	Burnout[1]: The 16-item Oldenburg Burnout Inventory (Demerouti et al., 2003). Thriving[2]: The 10-item Thriving at Work Scale (Porath et al., 2011). Supervisor's transformational leadership[2]: The 15-item

		key resource). The aim was to find out if all employees benefit in a similar way from transformational leadership.		method. All questionnaires had to be completed within a week.				German version of the Multifactor Leadership Questionnaire Form 5X-Short (MLQ)<br><u>Openness to experience[2]:</u><br>six bipolar adjective pairs from German short version MRS-30 of the MRS Inventory
Magnavita 2018 [77]; Italy	2015-2017	The study described a new way of proceeding with medical surveillance in an SME in Rome and reported the observations and the short-term results of an primary stress prevention intervention using participatory ergonomic groups ("Gruppo di Ergonomia	Intervention study	Within medical examination in 2015 and 2017 the workers completed questionnaires containing the ERI questionnaire and Goldberg's Anxiety and Depression scale. In 2017 the workers who had participated in the intervention were asked about the results.	SME in Rome<br>$n = 57$<br>N. Administrative and support service activities	2. Organization of work: working time; work process;<br>3. Social relations: Managers;<br>5. new forms of work	Work-related stress;<br>mental wellbeing (anxiety, depression)<br><br>(comparison before and after an intervention)	<u>Mental wellbeing[1]:</u><br>Goldberg's Anxiety and Depression scale<br><u>Work-related stress[1]:</u><br>The ERI questionnaire (the short, validated version in Italian)

		Partecipativa", GEP©).						
Sommovigo et al. 2018 [68]; Italy and Ireland	2016-2017	In this study similarities and differences between Italian and Irish workers' well-being in relation to robberies and theft exposure were investigated by examining post-traumatic symptoms and trauma-related coping self-efficacy.	Cross-sectional study	A self-developed, anonymous self-reported paper-and-pencil- or online questionnaire was used to collect data. The questionnaire contained previously used scales for some variables.	Small Italian businesses *n* = 319<br>Small Irish businesses *n* = 251<br>G. Wholesale and retail trade repair of motor vehicles and motorcycles	1. Work content and task: emotional demands	Main outcome: post-traumatic stress disorder (PTSD)<br><br>Secondary outcome: coping Self-efficacy	<u>PTSD[1]:</u><br>The six-item Impact of Event-Revised scale (IES-R)<br><u>Coping self-efficacy[1]:</u><br>The seven-item trauma-related coping self-efficacy scale (CSE-7)<br><u>Robbery and/or theft exposure[2]:</u><br>two dichotomous items and a check-list, to investigate peri-trauma variables among victims of robberies at work, proposed by Giorgi et al. (2015)
Setti et al. 2018 [95]; Italy	Unknown	It was investigated to what extent workers' mental health, coping-self-efficacy, social support seeking, workload and	Cross-sectional study	Respondents filled in an anonymous self-report questionnaire, containing previously used and validated scales; victims of	Small businesses *n* = 492<br>G. Wholesale and retail trade repair of motor vehicles and motorcycles<br>I. Accommodation and food service	1. Work content and task: emotional demands;<br>2. Organization of work: work process	Main outcome: general psychological health<br><br>Secondary outcomes: post-traumatic stress-symptoms;	<u>General psychological health[1]:</u><br>General Health Questionnaire (GHQ-12)<br><u>Post-traumatic stress symptoms[1]:</u><br>The six-item Impact

		job satisfaction was affected by experiencing robberies and/or thefts at work.		violence answered additional questions related to post-traumatic stress symptomatology, trauma related coping self-efficacy and use of mental health services.	activities		coping self-efficacy; social support seeking; job satisfaction; work load	of Event-Revised scale (IES-R) <u>Trauma-related coping self-efficacy (CSE)[1]:</u> Seven-item Coping self-efficacy scale (CSE-7) <u>Social support seeking[2]:</u> Coping Orientation to Problem Experienced scale, COPE-IV <u>Job satisfaction[2]:</u> single item <u>Workload[2]:</u> subscale taken from the Areas of Work life Survey
Magola et al. 2018 [78]; United Kingdom	2015-2016	The aim was to identify the challenges and their relative importance, novice community pharmacists' (NCPs) are faced at transition to independent practitioners.	Qualitative study	The nominal group technique (NGT), a data collection method which used group discussion, was used to identify the challenges faced by NCPs as they transition to independent.	Novice community pharmacists' (NCPs); independent practitioner *n* = 25 Q. Human health and social work activities	1. Work content and task: responsibility; qualification; 2. Organization of work: communication/ cooperation; 3. Social relations: colleagues	No outcome predefined	Nominal group technique (NGT) developed by Delbecq[1,2]

Yeh et al. 2018 [73]; Taiwan	2013	Employees' work stress, well-being and burnout in a newly industrialized country were investigated with regard to public-private and company size differences.	Cross-sectional study	Data of a national employee survey, conducted in 2013 by the Institute of Labor, Occupational Safety and Health (ILOSH) of the Taiwan government, was used for analysis.	Private SMEs < 200 employees<br>*n* = 10319;<br>C. Manufacturing;<br>F. Construction:<br>D. electricity, gas;<br>Service (not specified), several other sectors (1,2%)	1. Work content and task: freedom of action;<br>2. Organization of work: working time;<br>5. New forms of work	Burnout	Burnout[1]:<br>Chinese version of the Copenhagen Burnout Inventory (C-CBI)<br>Job characteristics[2]:<br>Chinese version of Job Content Questionnaire (C-JCQ)<br>Job instability[2]: four subjective and objective indicators (questions)
Rastogi et al. 2018 [96]; India	Unknown	The two alternative theoretical explanations of disengagement at work were tested: the relationship between job complexity and disengagement (according to job-demands-resources (JD-R) perspective) and the relationship between exhaustion and disengagement (according to the	Cross-sectional study	A self-developed and translated survey, containing previously used scales for some variables, was used for investigation.	Agro-processing unit 138 employees<br>*n* = 119;<br>C. Manufacturing	1. Work content and task Variability;<br>2. Organization of work: work process	Main outcome: disengagement<br><br>Secondary outcomes:<br>work exhaustion;<br>resilience;<br>job complexity	Disengagement[1]:<br>five items from the disengagement subscale of the Oldenburg Burnout Inventory (OLBI) (Demerouti et al., 2001)<br>Exhaustion[1]:<br>four items from the exhaustion subscale of the OLBI<br>Resilience[1]:<br>five items from Smith et al. (2008)<br>Job complexity[1]:<br>three items from Shaw and Gupta (2004)

		process model of burnout). Also the mediating role of Resilience (referring to the conservation of resources (COR) as an integrative framework) on both these relationships was examined.					
Estévez-Mujica et al. 2018 [97]; Italy	2018	The aim of this study was to identify employees at risk of job burnout, in an innovative way, by analyzing the e-mail communication patterns.	Cross-sectional study	Burnout data was collected via an online-survey. All internal company e-mail data (communications between company employees) were collected over a five-month period and analyzed.	Medium-sized company;2012 employees<br>*n* = 57;<br>M. Professional, scientific and technical activities	1. Work content and task: information/ supply of information;<br>2. Organization of work: work process; communication/ cooperation;<br>3. Social relations: colleagues	Dependent variable: burnout;<br><br>Independent variables: e-mail communication patterns:<br>1. volume variables<br>2. behavioral variables<br>3. positional variables	Burnout[1]:<br>online version of the OLBI questionnaire<br>E-volume variables[1]:<br>the sum of all e-mails sent or received; the average daily number of e-mails sent or received over the period of analysis; the total volume of e-mails sent or received, compared to a hierarchical level baseline; email bursts (sequence of e-mails that occurs within a specific

								time threshold between each e-mail)<br>Positional variables[1]:<br>UCINET's ego network unweighted basic measures procedure and UCINET's ego networks structural holes procedure<br>Behavioral variables[1]:<br>Ratio Sent/Received; E-mails Sent During Out-of-office Hours; Higher Hierarchical Level Reciprocity<br>Control variables[2]:<br>self-developed online questionnaire
Encrenaz et al. 2019 [30]; France	2010	The differences between enterprise sizes in relation to the psychosocial work environment and	Cross-sectional study	Data from a French periodical cross-sectional survey (2010 SUMER) were analyzed.	SMEs <250 employees; 73,2% of 31420 ≈ n = 23000; private companies, economic sector not reported	1. Work content and task: freedom of action; variability;<br>2. Organization of work: work process;<br>3. Social relations: managers	Depressive episodes; anxious episodes;	Depressive and anxious episodes[1]:<br>Hospital anxiety and depression scale (HADS)<br>Perceived working conditions[2]:<br>French version of

		psychological health was examined. The mediating role of perceived working conditions on anxious or depressive episodes was tested.						Job Content Questionnaire (JCQ)
Voss et al 2019 [98]; Germany	(1) 2016–2017<br>(2) 2018	The study assessed the accessibility and expected availability of workers in SMEs in order to identify starting points for workplace health management to improve the way these aspects are dealt with.	Two cross-sectional studies	Data from two surveys (2016/2017 and 2018) were analyzed using a self-developed questionnaire which was enhanced for the second survey.	(1) 11 SMEs;<br>(2) 1 SME<br>(1) $n = 1198$<br>(2) $n = 138$;<br>several economic sectors	5. New forms of work	Psychological stress due to permanent availability;<br><br>Secondary outcomes: frequency of contacts; expectations for replying	Self-developed questionnaire[1,2]

* For better readability, we refrained from indicating references for assessment instruments (e.g. questionnaires) used within the cited studies. Source references can be found in original literature.
References were only given in case when the cited study used methods for measurement taken from another study and this reference was cited by authors.
[1]Measurements of outcomes
[2]Measurements of confounders / mediators /control variables
