# Supplementary material for: Work-Related Psychosocial Stress in Small and Medium-Sized Enterprises: An Integrative Review
Source: Int J Environ Res Public Health. 2020 Oct 13;17(20):7446. doi: 10.3390/ijerph17207446 (PMC7650689; doi:10.3390/ijerph17207446)
Supplement: Supplementary file 1 [file ijerph-17-07446-s001.zip › Supplementary table S3 - Allocation to economic sectors.pdf]

Supplementary table S3. Allocation to economic sectors according to the International Standard Industrial Classification of All Economic Activities [51].

Section	Description	Reference	Total Number (n)
A.	Agriculture, forestry and fishing	[29]	1
B.	Mining and quarrying	---	0
C.	Manufacturing	[29, 31, 58, 61-65, 67, 71, 73, 74, 84, 87, 89, 94, 96]	17
D.	Electricity, gas, steam and air conditioning supply	[73]	1
E.	Water supply; sewerage, waste management and remediation activities	---	0
F.	Construction	[29, 67, 73, 77]	4
G.	Wholesale and retail trade; repair of motor vehicles and motorcycles	[59, 68, 77, 82, 95]	5
H.	Transportation and storage	[92]	1
I.	Accommodation and food service activities	[59, 66, 68]	3
J.	Information and communication	[31, 66, 75, 76]	4
K.	Financial and insurance activities	---	0
L.	Real estate activities	---	0
M.	Professional, scientific and technical activities	[58, 60, 97]	3
N.	Administrative and support service activities	[58, 77]	2
O.	Public administration and defense; compulsory social security	---	0
P.	Education	---	0
Q.	Human health and social work activities	[77, 78, 83, 85]	4
R.	Arts, entertainment and recreation	[31]	1
S.	Other service activities	[29, 71]	2
T.	Activities of households as employers; undifferentiated goods- and services-producing activities of households for own use	---	0
U.	Activities of extraterritorial organizations and bodies	---	0
Service (not specified from author)		[29, 71, 73]	3
Several sectors reported (i.e. nationwide surveys)		[30, 57, 70, 72, 73, 92, 93, 98]	8
No sector reported		[69, 79-81, 86, 88, 90]	7
